# Consolidative thoracic radiation therapy for extensive-stage small cell lung cancer in the era of first-line chemoimmunotherapy: preclinical data and a retrospective study in Southern Italy

**DOI:** 10.3389/fimmu.2023.1289434

**Published:** 2024-01-18

**Authors:** Vito Longo, Carminia Maria Della Corte, Alessandro Russo, Francesca Spinnato, Francesca Ambrosio, Riccardo Ronga, Antonella Marchese, Teresa Del Giudice, Concetta Sergi, Francesca Casaluce, Marina Gilli, Michele Montrone, Valerio Gristina, Vincenzo Sforza, Maria Lucia Reale, Raimondo Di Liello, Alberto Servetto, Helga Lipari, Claudio Longhitano, Laura Vizzini, Anna Manzo, Antonella Cristofano, Loretta Paolelli, Annalisa Nardone, Simona De Summa, Antonella Perrone, Carmela Bisceglia, Caterina Derosa, Valerio Nardone, Giuseppe Viscardi, Domenico Galetta, Fabiana Vitiello

**Affiliations:** ^1^Medical Thoracic Oncology Unit, IRCCS Istituto Tumori ”Giovanni Paolo II“, Bari, Italy; ^2^Department of Precision Medicine, University of Campania Luigi Vanvitelli, Naples, Italy; ^3^Department of Hematology-Oncology, Papardo Hospital, Messina, Italy; ^4^UOC Oncologia Medica Ospedali Riuniti Villa Sofia Cervello, Palermo, Italy; ^5^UOC Oncologia AORN Cardarelli, Hospital Antonio Cardarelli, Naples, Italy; ^6^Ospedale La Maddalena, Palermo, Italy; ^7^Medical Oncology Unit, AOU Renato Dubecco De Lellis Hospital, Catanzaro, Italy; ^8^UOC Oncologia ARNAS Garibaldi Catania, Azienda Sanitaria Provinciale di Catania, Catania, Italy; ^9^Divison of Medical Oncology, AORN S.G. Moscati Hospital (San Giuseppe Moscati Hospital of National Importance and High Specialty), Avellino, Italy; ^10^Department of Pulmonary Oncology, AORN Azienda Ospedaliera dei Colli Monaldi, Naples, Italy; ^11^Department of Surgical, Oncological and Oral Sciences, University of Palermo, University of Palermo, Palermo, Italy; ^12^Oncologia Clinica Sperimentale Toraco-Polmonare, G. Pascale National Cancer Institute Foundation (IRCCS), Naples, Italy; ^13^Medical Oncology Unit, Vito Fazzi Hospital, Lecce, Italy; ^14^Oncology Unit Ospedale del Mare, ASL Napoli 1, Napoli, Italy; ^15^Department of Clinical Medicine and Surgery, School of Medicine and Surgery, University of Naples Federico II, Naples, Italy; ^16^Oncologia Ospedale Cannizzaro Catania, Medical Oncology Unit, Cannizzaro Hospital, Catania, Italy; ^17^UOC Oncologia Ospedale Maria Paterno Arezzo (OMPA), Ragusa, Italy; ^18^UOC Oncology Agrigento Health Authority, Agrigento, Italy; ^19^Dipartimento di Oncologia e Oncoematologia, Ospedale Generale Regionale F. Miulli, Acquaviva, Italy; ^20^Unitá Opertiva Complessa di Radioterapia, I.R.C.C.S. Istituto Tumori “Giovanni Paolo II”, Bari, Italy; ^21^Molecular Diagnostics and Pharmacogenetics Unit, IRCCS Istituto Tumori “Giovanni Paolo II”, Bari, Italy

**Keywords:** consolidative radiotherapy, SCLC - small cell lung cancer, chemoimmunotherapy, innate immunity, safety

## Abstract

**Background:**

Consolidative thoracic radiotherapy (TRT) has been commonly used in the management of extensive-stage small cell lung cancer (ES-SCLC). Nevertheless, phase III trials exploring first-line chemoimmunotherapy have excluded this treatment approach. However, there is a strong biological rationale to support the use of radiotherapy (RT) as a boost to sustain anti-tumor immune responses. Currently, the benefit of TRT after chemoimmunotherapy remains unclear. The present report describes the real-world experiences of 120 patients with ES-SCLC treated with different chemoimmunotherapy combinations. Preclinical data supporting the hypothesis of anti-tumor immune responses induced by RT are also presented.

**Methods:**

A total of 120 ES-SCLC patients treated with chemoimmunotherapy since 2019 in the South of Italy were retrospectively analyzed. None of the patients included in the analysis experienced disease progression after undergoing first-line chemoimmunotherapy. Of these, 59 patients underwent TRT after a multidisciplinary decision by the treatment team. Patient characteristics, chemoimmunotherapy schedule, and timing of TRT onset were assessed. Safety served as the primary endpoint, while efficacy measured in terms of overall survival (OS) and progression-free survival (PFS) was used as the secondary endpoint. Immune pathway activation induced by RT in SCLC cells was explored to investigate the biological rationale for combining RT and immunotherapy.

**Results:**

Preclinical data supported the activation of innate immune pathways, including the STimulator of INterferon pathway (STING), gamma-interferon-inducible protein (IFI-16), and mitochondrial antiviral-signaling protein (MAVS) related to DNA and RNA release. Clinical data showed that TRT was associated with a good safety profile. Of the 59 patients treated with TRT, only 10% experienced radiation toxicity, while no ≥ G3 radiation-induced adverse events occurred. The median time for TRT onset after cycles of chemoimmunotherapy was 62 days. Total radiation dose and fraction dose of TRT include from 30 Gy in 10 fractions, up to definitive dose in selected patients. Consolidative TRT was associated with a significantly longer PFS than systemic therapy alone (one-year PFS of 61% vs. 31%, p<0.001), with a trend toward improved OS (one-year OS of 80% vs. 61%, p=0.027).

**Conclusion:**

Multi-center data from establishments in the South of Italy provide a general confidence in using TRT as a consolidative strategy after chemoimmunotherapy. Considering the limits of a restrospective analysis, these preliminary results support the feasibility of the approach and encourage a prospective evaluation.

## Introduction

Small cell lung cancer (SCLC) is a unique subtype of lung cancer with an aggressive behavior and dismal prognosis. Approximately two-thirds of patients have extensive stage (ES)-SCLC at the diagnosis, with a five-year survival rate of <3.0% (1).

Consolidative thoracic radiotherapy (TRT) has demonstrated a two-year overall survival (OS) increase in ES-SCLC patients who were responsive to first-line platinum-based chemotherapy, thus becoming a tool utilized in clinical practice (2). Neither the phase III IMpower 133 (3) nor the phase III Caspian (4) explored TRT in ES-SCLC patients treated with chemotherapy plus immunotherapy. Furthermore, the more recent Asian trials concerning first-line chemoimmunotherapy, namely, CAPSTONE-1 (5) and ASTRUM005 (6), excluded this therapeutic approach. However, preliminary data show that TRT is well-tolerated in ES-SCLC patients treated with chemoimmunotherapy. Welsh et al. (7) assessed the safety of combining pembrolizumab with TRT, observing no grade 4–5 adverse events (AEs) and noting that only 6% of patients experienced grade 3 AEs. RAPTOR (NCT04402788) is an ongoing phase III clinical trial evaluating the addition of TRT to atezolizumab in ES-SCLC patients with a partial response (PR)/stable disease (SD) after standard chemotherapy plus atezolizumab (8). While prospective data are being collected, preclinical and retrospective studies represent a useful tool to guide clinical practice. Using a preclinical SCLC model with H82 and H524 cells and variable basal levels of STING, the present study demonstrated the activation of innate immune pathways after chemo- and radiotherapy. A multi-center retrospective study was performed to characterize the outcomes and toxicities of TRT in real-world ES-SCLC patients treated with first-line chemoimmunotherapy.

## Materials and methods

Data for patients with treatment-naive ES-SCLC were retrospectively collected at 16 Southern Italy cancer centers between June 2020 and April 2023. All patients were treated with four cycles of chemoimmunotherapy followed by immunotherapy maintenance with or without thoracic radiation. Patients with disease progression after four cycles of chemoimmunotherapy or those with disease progression prior to completion of two maintenance immunotherapy cycles were excluded. A total of 120 patients with ES-SCLC treated with chemoimmunotherapy were selected, of which 59 patients underwent TRT.

Indication for TRT as a consolidative treatment after chemoimmunotherapy and the time interval between the fourth chemoimmunotherapy cycle and mediastinal RT onset were assessed. The following patient characteristics were analyzed: age, gender, and presence of hepatic, cerebral, bone, and other metastases at the diagnosis (**Table 1**).

**Table 1:** Baseline patient characteristics.

Characteristics	No TRT, N = 59^1^	TRT, N = 59^1^	p-value^2^
Median age	71 (65, 75)	64 (60, 69)	<0.001
Sex			
Female	22 (37%)	26 (44%)	
Male	37 (63%)	33 (56%)	
Metastatic sites			
Lung			0.7
No	4 (6.8%)	5 (8.5%)	
Yes	55 (93%)	54 (92%)	
Liver			0.6
No	47 (80%)	48 (81%)	
Yes	12 (20%)	11 (19%)	
Brain			>0.9
No	50 (85%)	49 (83%)	
Yes	9 (15%)	10 (17%)	
Bone			0.8
No	42 (71%)	41 (69%)	
Yes	17 (29%)	18 (31%)	
Adrenal gland			0.7
No	48 (81%)	47 (80%)	
Yes	11 (19%)	12 (20%)	
Lymph nodes			0.026
No	44 (75%)	32 (54%)	
Yes	15 (25%)	27 (46%)	

^1^n (%); Median (IQR);

^2^Pearson’s Chi-squared test; Fisher’s exact test.

Considering the lack of data in the registry studies, the decisions to initiate TRT were assessed by the interdisciplinary tumor board. Mediastinal RT as consolidative treatment was offered in cases of the primary tumor response and the presence of a responsive or stable metastatic burden after four cycles of chemoimmunotherapy. All patients included in the analysis achieved a PR after four cycles of chemoimmunotherapy. The response types had to be deemed appropriate for TRT by the physician. At the same time, the decision whether to administer TRT was guided by the physician based on the opinion regarding TRT use in patients with ES-SCLC treated with chemoimmunotherapy. The gross target volume included the residual thoracic disease and positive lymph nodes. The clinical target volume included gross target volume in addition to a 5-mm margin and nodal region involved before the start of chemoimmunotherapy. The planning target volume included clinical target volume in addition to a 3-mm margin. However, a high variability in RT planning and dosimetry was reported among the different centers, ranging from patients receiving a radiation dose of 30 Gy in 10 fractions to patients undergoing hyperfractionated treatment. Treatment details concerning radiation regimen, such as total radiation dose, fraction, and biologically effective dose (BED), as well as chemotherapy schedules are summarized in **Table 2**.

**Table 2:** Treatment characteristics.

Grade of adverse events	TRT group (59 patients)				Non-TRT group (61 patients)			
	Number of events				Number of events			
	G1	G2	G3	G4-5	G1	G2	G3	G4-5
Fatigue		1				1		
Radiation dermatitis	1							
Rash	1	1						
Cough	1							
Pruritus	2				1			
Stomatitis					1			
Pneumonitis		4				2		
Radiation esophagitis		2						
Transaminase increase	1	2	1		1			
Thyroiditis		1						
Anemia	2				2			
Piastrinopenia						1		
Increase of creatinine					1			
Arthritis							1	
Gastrointestinal							1	

The present study was approved by the local Ethics Committee of Istituto Tumori Giovanni Paolo II in Bari (prot. n. 1223 CE) and conducted in accordance with the international standards of good clinical practice.

### Statistical analysis

Propensity score matching (PSM) and survival analyses and tests for equality of proportions were performed using the R v.3.6.3 environment. Propensity score matching was carried out using the “MatchIt” R package. Kaplan–Meier curves and Cox hazard regression analyses were implemented via the “survival” package. The Mantel–Cox test used to compare the Kaplan–Meier curves was done using the “survminer” package, while the “ggplot2” package was utilized to depict survival curves. To perform PSM, the used factors were gender, age, and site of metastasis (lung, liver, brain, bone, adrenal gland, and lymphonode).

## Results

### Preclinical data

Based on the published data defining a subgroup of SCLC patients, where responders to immunotherapy were classified as “inflamed” (9, 10) patients that express high levels of innate immune pathway genes, such as the STING pathway, and other preclinical data supporting STING levels as potential biomarkers of a response to combination chemo- and immunotherapy (11, 12), it was hypothesized that activation of the innate immune pathways may represent a tool that can be used to investigate anti-tumor immune activity *in vitro*.

**Figure 1:**
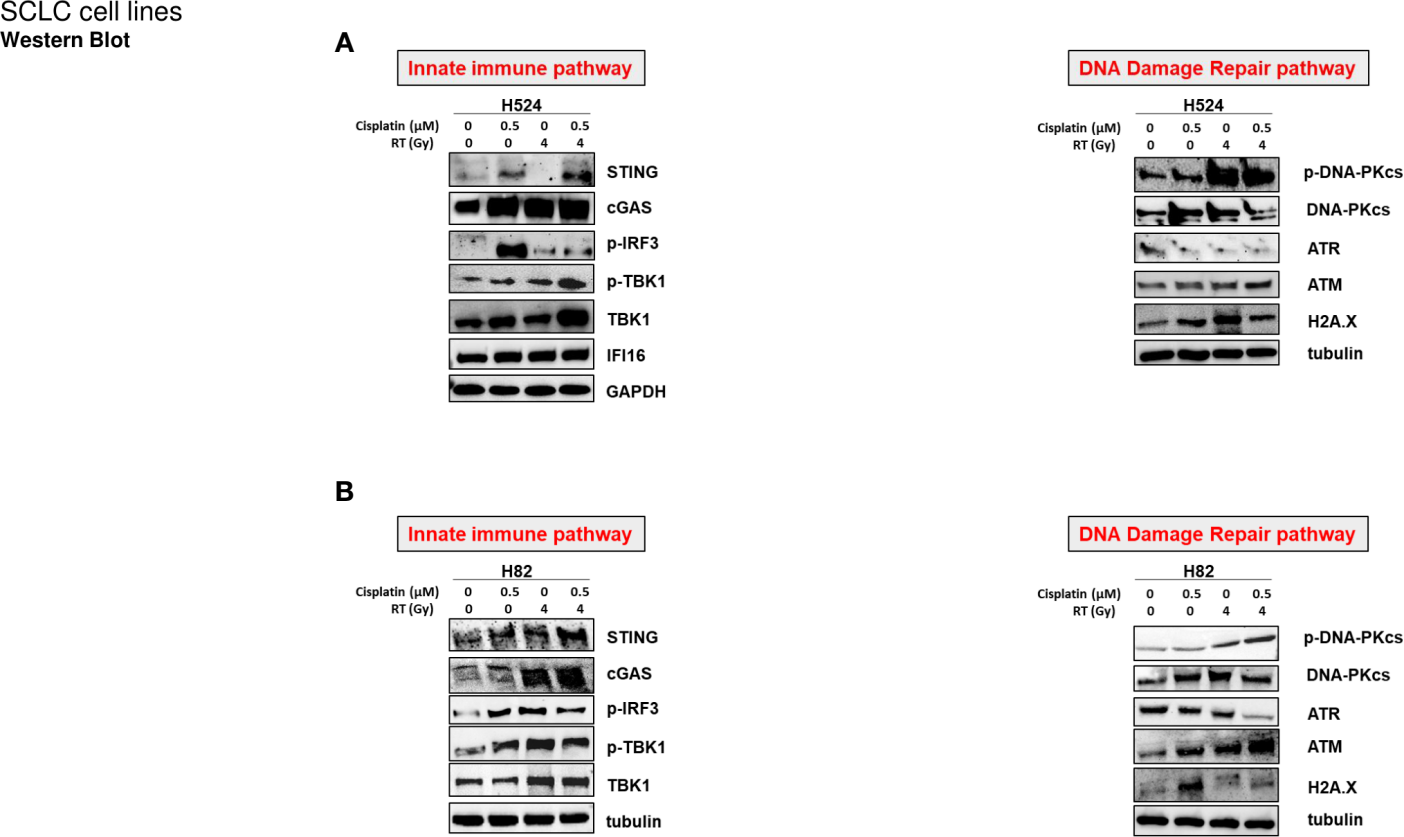
**(A)** H524 and **(B)** H82 SCLC cell lines were treated with or without cisplatin (0.5 µM) for 72 h, irradiated at 4 Gy (or not irradiated), and incubated for 24h Cell lysates were immunoblotted with the indicated antibodies (see **Supplementary Material**) to detect STING-mediated innate immune activation and DNA damage repair pathways. GAPDH and tubulin were used to ensure equal loading.

**Figure 2:**
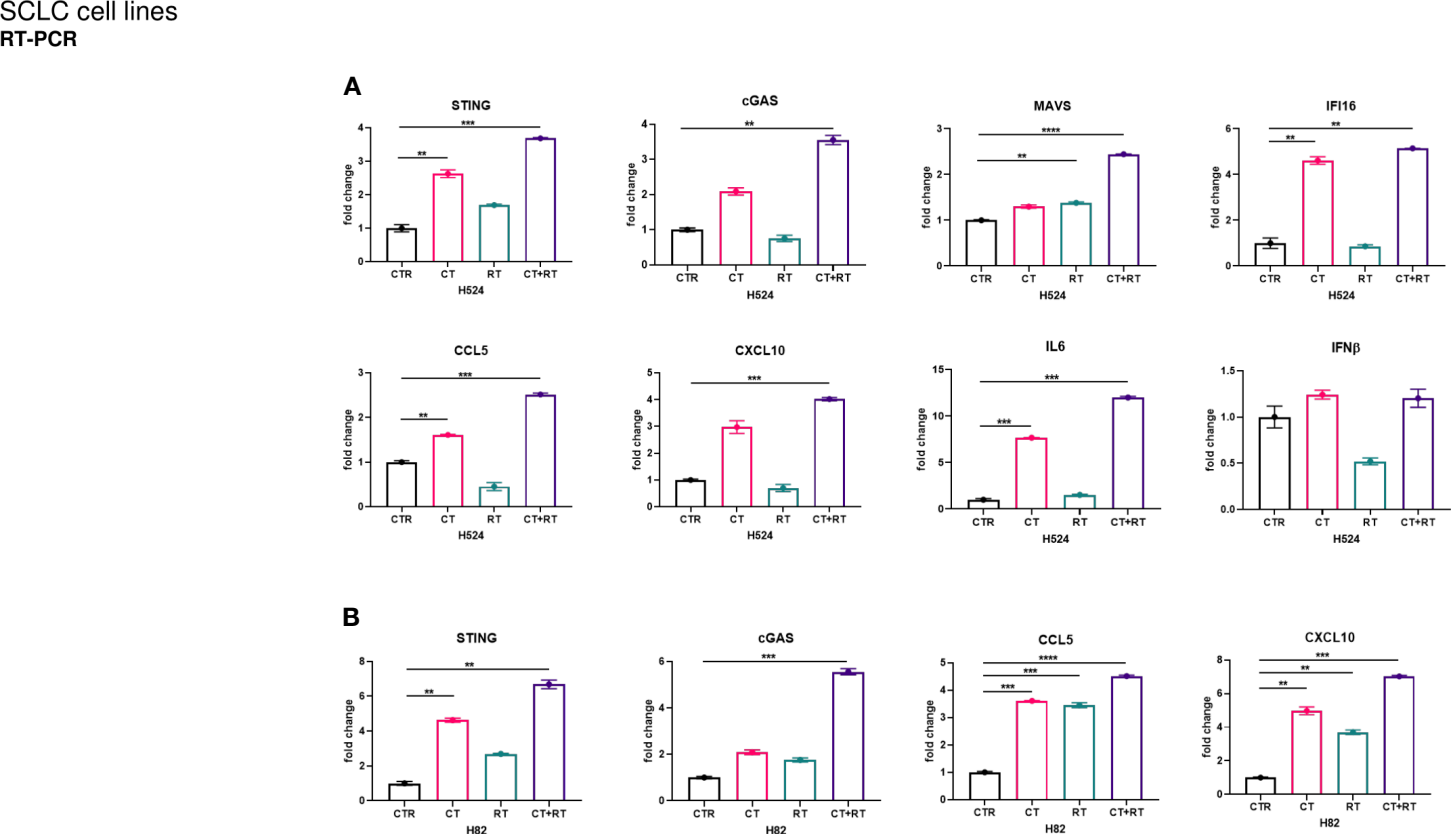
Both **(A)** H524 and **(B)** H82 SCLC cells were treated with cisplatin (CT; 0.5 µM) for 72 h and then irradiated at 4 Gy (RT). After irradiation, both cell groups (1 × 10^4^ cells/well) were cultured for 24 h and mRNA expression levels of innate immune activation markers in both SCLC cell lines were determined by quantitative real-time PCR (see **Supplementary Material**). Normalized expression data are represented as mean ± s.e.m. derived from three technical replicates calculated using the comparative method of 2-^(ΔΔCt) (reference gene 18S). One-tailed unpaired Student’s t-test with 95% confidence interval (CI), where ****P<0.0005, ***P<0.005, and **P< 0.05.

Two SCLC cell models of H82 and H524 cells with variable basal levels of STING (as examined by western blot and RT-PCR for protein and gene expression) were selected to perform sequential *in vitro* treatment with cisplatin and RT at the indicated doses. Changes in protein levels in the STING pathway members were investigated as an indicator of a response to DNA damage induced by chemotherapy and RT. In particular, cGAS, STING, and downstream proteins phosphor-TBK1 and phosphor-IRF3 were upregulated by cisplatin and RT in both cell lines (**Figures 1A, B**). Interestingly, these changes were accompanied by high levels of DNA damage as suggested by increased levels of H2AXM and other DNA damage-related proteins, such as ATM, ATR, and DNA-PK (**Figures 1A, B**). To further confirm STING pathway activation and to extend the investigation to another known sensor of DNA and RNA release in response to chemotherapy and RT, protein and RNA levels of DNA sensor IFI16 were shown to be increased using western blot analysis (**Figure 1**) and RT-PCR (**Figure 2**). In addition, the levels of RNA sensor MAVS were statistically significantly increased after sequential treatment with chemotherapy and RT, while inflammatory T cells recruited chemokines IL6, IFN-beta, CXCL5, and CXCL10 (**Figure 2**). RNA expression of STING and CGAS was also significantly increased (**Figure 2**), thus confirming that chemotherapy and RT sustain a direct activation of this innate pathway.

### Patient characteristics

A total of 120 patients with ES-SCLC were eligible for analysis. Only the patients who achieved an objective response were considered suitable for TRT after chemoimmunotherapy, while all patients with disease progression prior to completion of two maintenance immunotherapy cycles were excluded. A total of 59 patients underwent TRT. Patient characteristics are detailed in **Table 1**. Most of the patients were male, representing 56% of the TRT group and 63% of the non-TRT group. The median age was lower in the TRT group than in the non-TRT group at 64 years vs. 71 years (p<0.001), respectively. Lymph node involvement was higher in the TRT group at 46% compared to 25% in the non-TRT group (p=0.021). Non-significant differences in brain, bone, and liver metastases were described. Time of TRT onset after four cycles of chemoimmunotherapy was ≥60 days in 31 patients. RT dose was highly variable among different centers, ranging from 30 Gy to bifractionated radiation of 45 Gy in some patients. Overall, 54% (32) of patients received an RT dose of <45 Gy (**Table 2**).

### Patient outcomes and toxicity

Propensity score matching was used to estimate the RT effect on survival. One-to-one nearest neighbor matching was used. All patients who underwent RT (n=59) were matched to those who did not, reaching a good balance with all standardized mean differences below 0.1 after matching excluded only two RT-untreated subjects. Matched data were then used for survival analyses.

**Figure 3:**
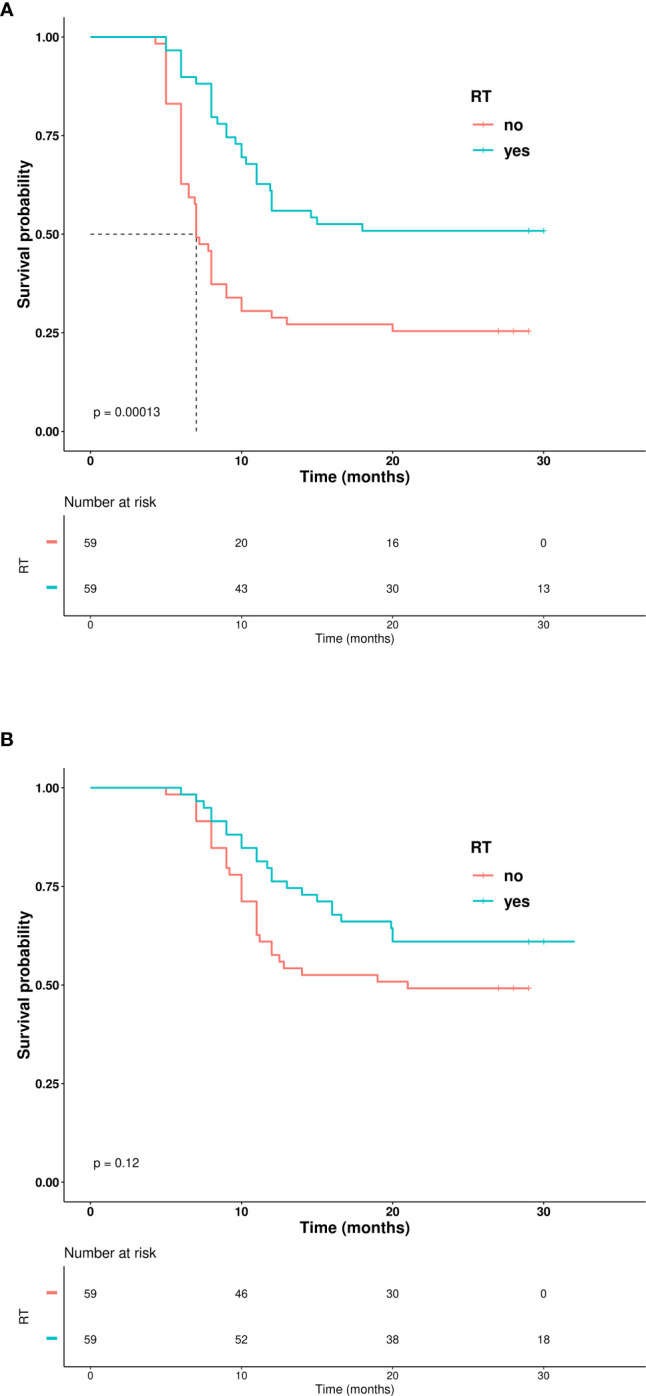
**(A)** Kaplan–Meier curves showing progression-free survival. **(B)** Kaplan–Meier curves showing overall survival.

**Figure 4:**
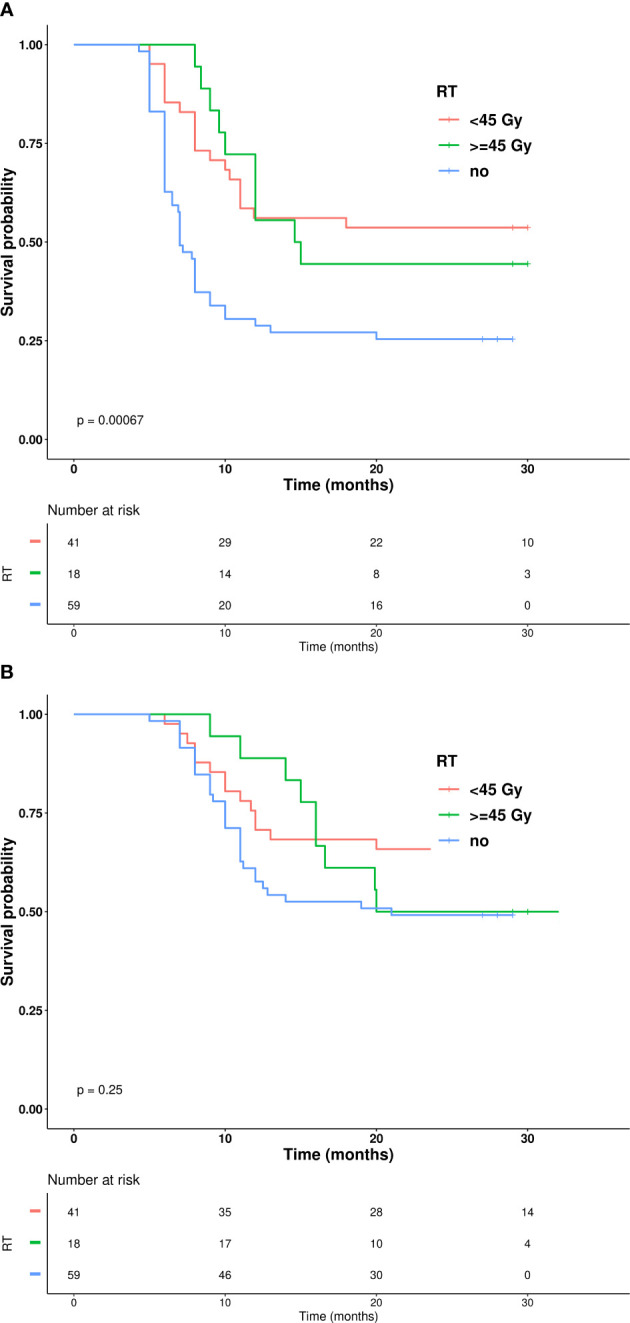
**(A)** Kaplan–Mier curves showing progression free survival based on radiation BED. **(B)** Kaplan–Mier curves showing overall survival based on radiation BED.

Kaplan–Meier curves representing PFS and OS for patients receiving TRT vs. patients treated only with chemoimmunotherapy are depicted in **Figures 3A, B**. PFS was significantly longer in the TRT group, with one-year PFS of 61% compared to 31% in the non-TRT group (p<0.0001). The OS analysis demonstrated a trend toward improvement (one-year OS of 80% vs. 61%, p=0.027). The AE grade and incidence are listed in **Table 3**. Of the 59 patients treated with TRT, only 10% experienced radiation toxicity (four cases of G2 radiation-induced pneumonitis; two cases of G2 radiation-induced esophagitis), while no ≥G3 radiation-induced AEs occurred. Only one G3 AE was reported in the TRT group (transaminase increase) compared to two G3 AEs in the non-TRT group (one case each of arthritis and gastrointestinal toxicity). No G4-G5 AEs were observed in the present study. Finally, the RT-treated group was further stratified according to RT dose (RT<45 Gy and RT≥45 Gy), and pairwise Kaplan–Meier curve analysis showed no statistical difference in outcome in the two subgroups of RT-treated patients (**Figures 4A, B**).

**Table 3:** Safety data summary.

Treatment characteristics	No, N = 61	Yes, N = 59
Chemoimmunotherapy schedule		
•Carboplatin-etoposide-Atezolizumab •Carboplatin-etoposide-Durvalumab •Cisplatin-etoposide-Durvalumab	60 (98.3%) 1 (1.7%)	54 (91.5%) 3 (5.1%) 2 (3.4%)
Radiotherapy		
•Total radiation dose, fraction, BED: 45 Gy, 2 daily fractions of 1.5 Gy, 51.75 Gy 60 Gy, 2 Gy x 30, 72 Gy 39 Gy, 3 Gy x 13, 50,7 Gy 36 Gy, 3 Gy x 12, 46,8 Gy 50.4 Gy, 2.78 Gy x 18, 63.95 Gy 30 Gy, 3 Gy x 10, 39 Gy 20 Gy, 4 Gy x 5, 28 Gy		5 (8.5%) 11 (18.6%) 6 (10,2%) 6 (10.2%) 14 (23.7%) 15 (25.4%) 2 (3.4%)

## Discussion

TRT demonstrated a two-year OS increase in ES-SCLC patients responsive to first-line platinum-based chemotherapy (2). However, recent chemoimmunotherapy trials have not explored this therapeutic approach. Preliminary data show a favorable safety profile and prognosis improvement in ES-SCLC patients receiving first-line PD-L1 inhibitors combined with chemotherapy (13, 14). In accordance, current ASTRO, ASCO, and a recent Canadian consensus express a favorable opinion concerning TRT, especially in patients with residual mediastinal tumors and low extra-thoracic burden (15, 16).

Concerning the efficacy data, many studies have demonstrated a synergism between RT and immunotherapy, known as the abscopal effect, due to tumor antigen release after tumor cell irradiation (17). These studies are principally related to non-small cell lung cancer. Less data concerning SCLC are available, probably due to more recent use of immunotherapy to treat this disease. Specifically, no biomarkers are available for identifying SCLC patient candidates to be good responders to chemo-immunotherapy. Translational studies have identified transcriptomic definitions of SCLC subgroups (9), highlighting that the subgroup of patients classified as “inflamed” over-expresses innate immune pathway genes, such as the STING pathway genes *CCL5* and *CXCL10*, that sustain the inflamed microenvironment primed for immunotherapy response. Since other studies demonstrated that DNA damage induction with chemotherapy and RT (12) stimulates the STING pathway and the immune response, the present study used preclinical SCLC cell models to show a significant positive modulation of innate immune pathway genes (STING and MAVS) accompanied by increased DNA damage with sequential chemotherapy and RT treatment *in vitro*. These data suggest that this strategy may amplify the immune response in patients. Unfortunately, the preclinical *in vitro* data may not allow for consideration of the impact of the immune microenvironment, thus providing only a partial estimate of the interactions between RT and immune response.

Currently, the absence of prospective data makes it necessary to analyze the available retrospective evidence. A monocentric case series evaluating 23 ES-SCLC patients treated with TRT during immunotherapy maintenance reported OS and PFS improvement (18). Accordingly, survival improvement was noted in a more extensive cohort in a study by Peng et al. (14) that included 70 patients treated with TRT. In particular, median PFS and OS were significantly longer in patients who received TRT compared to those who did not, with median PFS of 9.5 months and 7.2 months, respectively, (hazard ratio (HR) of 0.59; p=0.009) and median OS of 24.1 months and 18.5 months, respectively (HR=0.53; p=0.016). Nevertheless, a recent study (13) evaluating a cohort of 100 patients, where 47 of participants were treated with TRT, noted no improvement in PFS and OS. On the other hand, TRT increased local RFS, resulting in a significant improvement in quality of life. Similar to another retrospective study, the present investigation demonstrated a TRT benefit in a cohort of 120 patients treated with first-line chemotherapy plus immunotherapy, where 59 patients also received TRT. A significantly longer PFS was shown compared to systemic therapy alone (one-year PFS of 61% vs. 31%, p<0.0001), as well as a trend toward improved OS (one-year OS of 80% vs. 61%, p=0.027). In order to minimize selection bias, all patients with disease progression prior to completion of two maintenance immunotherapy cycles were excluded. Moreover, metastatic site patterns were similar between the two groups, except for a higher rate of lymph node involvement in the TRT group. Only 19% of the TRT group had liver involvement. Secondary analysis described in the CREST trial (19) suggested that hepatic metastases are an important factor for stratifying patients who might benefit from TRT. However, the present TRT group had a lower median age. At the same time, the retrospective nature of the data collected in the present and other studies resulted in a high heterogeneity in patient characteristics and RT schedules. Therefore, data from prospective trials are needed. Currently, there are two ongoing trials assessing the benefit of TRT in patients with ES-SCLC. The RAPTOR trial (8) is randomizing patients based on standard immunotherapy maintenance or maintenance with consolidative radiation to up to five thoracic and/or extrathoracic sites. While, the TREASURE trial (20) evaluating the benefit of TRT in patients without brain metastases, recently closed the recruitment due to high number of grade 5 AEs, even if these AEs are not clearly tied to either immunotherapy or TRT.

With regard to toxicity, low AE rates have been reported in prior published retrospective studies (13, 18, 21). Only Peng et al. (14) described higher rates of treatment-related pneumonitis of 35.1% and 15.8% in the TRT and non-TRT groups (p=0.018), respectively, including one death due to immune checkpoint inhibitors related pneumonitis. In the present study, pneumonitis occurred in four (6.5%) and two (3.4%) patients in the TRT and non-TRT group, respectively. In contrast to the Peng et al. study, all of the pneumonitis cases described in our study were of grade 2. Only one G3 AE was reported in the TRT group compared to two G3 AEs in the non-TRT group. Moreover, no G4–G5 AEs were observed in the present study, which was similar to other reports (13, 18). A good safety profile for TRT in SCLC patients treated with chemoimmunotherapy was also noted.

## Conclusions

A significant positive modulation of the innate immune pathway genes (STING and MAVS) was demonstrated in SCLC cell models using sequential chemotherapy and RT treatments *in vitro*, suggesting a possible role of TRT in ES-SCLC patients treated with chemoimmunotherapy.

Multi-center data from Southern Italy provide a general confidence in using TRT as a consolidative strategy after chemoimmunotherapy based on a clinical multidisciplinary decision. With the limit of a retrospective analysis, these preliminary results support the feasibility of the approach and encourage a prospective evaluation.

## Data availability statement

The raw data supporting the conclusions of this article will be made available by the authors, without undue reservation.

## Ethics statement

The studies involving humans were approved by Istituto Tumori Giovanni Paolo II Bari. The studies were conducted in accordance with the local legislation and institutional requirements. The participants provided their written informed consent to participate in this study. Written informed consent was obtained from the individual(s) for the publication of any potentially identifiable images or data included in this article.

## Author contributions

VL: Conceptualization, Data curation, Supervision, Writing – original draft, Writing – review & editing, Investigation, Methodology. CDC: Conceptualization, Funding acquisition, Supervision, Validation, Writing – review & editing, Resources, Visualization, Software. AR: Data curation, Investigation, Methodology, Supervision, Writing – review & editing. FS: Data curation, Investigation, Writing – review & editing. FA: Data curation, Investigation, Writing – review & editing. RR: Data curation, Investigation, Writing – review & editing. AMar: Data curation, Investigation, Writing – review & editing. TG: Data curation, Investigation, Writing – review & editing. CS: Data curation, Investigation, Writing – review & editing. FC: Data curation, Investigation, Writing – review & editing. MG: Data curation, Investigation, Writing – review & editing. MM: Data curation, Investigation, Writing – review & editing. VG: Data curation, Investigation, Methodology, Writing – review & editing. VS: Data curation, Investigation, Writing – review & editing. MR: Data curation, Investigation, Writing – review & editing. RL: Data curation, Investigation, Writing – review & editing. AS: Data curation, Investigation, Writing – review & editing. HL: Data curation, Investigation, Writing – review & editing. CL: Data curation, Investigation, Writing – review & editing. LV: Data curation, Investigation, Writing – review & editing. AMan: Data curation, Investigation, Writing – review & editing. AC: Data curation, Investigation, Writing – review & editing. LP: Data curation, Investigation, Writing – review & editing. AN: Conceptualization, Data curation, Investigation, Methodology, Project administration, Writing – review & editing. SS: Conceptualization, Data curation, Formal analysis, Investigation, Methodology, Writing – review & editing. AP: Data curation, Formal analysis, Investigation, Software, Writing – review & editing. CB: Data curation, Formal analysis, Investigation, Project administration, Writing – review & editing. CD: Data curation, Formal analysis, Investigation, Methodology, Software, Writing – review & editing. VN: Conceptualization, Investigation, Methodology, Software, Writing – review & editing. GV: Data curation, Investigation, Writing – review & editing. DG: Supervision, Writing – review & editing. FV: Conceptualization, Data curation, Investigation, Methodology, Project administration, Resources, Supervision, Validation, Visualization, Writing – review & editing.
