# Supplementary material for: Consolidative thoracic radiation therapy for extensive-stage small cell lung cancer in the era of first-line chemoimmunotherapy: preclinical data and a retrospective study in Southern Italy
Source: Front Immunol. 2024 Jan 18;14:1289434. doi: 10.3389/fimmu.2023.1289434 (PMC10830694; doi:10.3389/fimmu.2023.1289434)
Supplement: Supplementary file 1 [file DataSheet_1.docx]

**Materials and Methods**

***Reagents*** – Primary antibodies for western blot analysis against MAVS (D54A9E) (24930, 1:1000), STING (D2P2F) (13647, 1:1000), phospho-DNA-PKcs (Ser2056) (E9J4G) (68716, 1:1000), DNA-PKcs (E6U3A) (38168, 1:1000), IFI16 (D8B5T) (14970, 1:1000), phospho-TBK1/NAK (Ser172) (D52C2) (5483, 1:1000), TBK1/NAK (E8I3G) (38066, 1:1000), phospho-IRF-3 (Ser386) (E7J8G) (37829, 1:1000), cGAS (D1D3G) (15102, 1:1000), Histone H2A.X (2595), GAPDH (14C10) (2118, 1:1000), were purchased from Cell signaling (Danvers, MA) and α-Tubulin (AA13, 1:2000) from Sigma Chemical Co. Cisplatin was purchased from Selleck chemicals (Houston, TX).

***Cell lines* –** SCLC cell lines NCI-H524 (ATCC Cat#CRL-5831; RRID: CVCL_1568) and NCI-H82 (ATCC Cat#HTB-175; RRID: CVCL_1591) were maintained in RPMI 1640 (Sigma-Aldrich, R8758) supplemented with 10% FBS (Sigma-Aldrich) and 1× penicillin–streptomycin (Sigma-Aldrich, P0781) in a humidity-controlled environment (37 °C, 5% CO_2_). Cell lines were obtained from the American Type Culture Collection (ATCC). The morphology of the cell lines was monitored, and the cell lines were routinely tested for Mycoplasma with a mycoplasma detection kit (InvivoGen).

***RNA extraction and cDNA synthesis –*** Total RNA was obtained from cell lines using TRIsure reagent (Meridian Bioscience, BIO-38033). RNA concentrations were measured with a Nanodrop 2000 spectrophotometer (Thermo Fisher Scientific). After RNA extraction, cDNA was generated from 500 ng of total RNA using a SensiFAST cDNA Synthesis Kit (Meridian Bioscience, BIO-65053) at the following conditions: 25 °C for 10 min, 42 °C for 15 min, 85 °C for 5 min.

***Gene expression analysis by quantitative reverse transcription-polymerase chain reaction (qRT-PCR) –*** mRNA expression levels of STING, MAVS, IFI16, cGAS, CCL5, CXCL10, IL6, IFNβ genes were evaluated by qRT-PCR with a QuantStudio 7-Flex (Applied Biosystems) using the SensiFAST SYBR Hi-ROX Kit (Meridian Bioscience, BIO-92005) and the following conditions: 50 °C for 2 min (stage 1) followed by a denaturation step at 95 °C for 10 min (stage 2) and then 40 cycles at 95 °C for 15 s and 60 °C for 1 min (stage 3). All samples were run in duplicate, in 20 μL reactions and relative expression of genes was determined by normalizing to 18S, used as internal control gene; to calculate relative gene expression in value it was used the 2- ΔCt or 2- ΔΔCt method. Non-specific signals caused by primer dimers were excluded by dissociation curve analysis and use of non-template controls.

***Table S1. Primer sequences used for qRT-PCR analysis***

| Gene | Primer |
| --- | --- |
| STING | 5ʹ-CCTGTTGCTGCTGTCCATCT-3ʹ  5ʹ-ATGTTCAGTGCCTGCGAGAG-3ʹ |
| cGAS | 5ʹ-CTCCACGAAGCCAAGACCTC-3ʹ  5ʹ-GCGGCTGAGCTTCAACTTCT-3ʹ |
| MAVS | 5′-CTTCCCCTGTGTTCACCTTTCTG-3′  5′-CATTGTCCCCTGGG-TCCTTCA-3’ |
| IFI16 | 5′-ACAAACCCGAGAAACAATGACC-3′  5′-GCATCTGAGGAGTCCGAAGA-3′ |
| CCL5 | 5’-CCTGCTGCTTTGCCTACATTGC-3’  5’-ACACACTTGGCGGTTCTTTCGG-3’ |
| CXCL10 | 5'- AGCAGAGGAACCTCCAGTCT -3'  5'- ATGCAGGTACAGCGTACAGT -3' |
| IL6 | 5’-ACTCACCTCTTCAGAACGAATTG-3’  5’-CCATCTTTGGAAGGTTCAGGTTG-3’ |
| IFNβ | 5'- AGTAGGCGACACTGTTCGTG -3'  5'- GCCTCCCATTCAATTGCCAC -3' |

***Western blot analysis* –** Protein lysates from SCLC cells were obtained by homogenization in RIPA lyses buffer [0.1% sodium dodecylsulfate (SDS), 0,5% deoxycholate, 1% Nonidet, 100 mmol/L NaCl, 10 mmol/L Tris–HCl (pH 7.4), 0.5 mmol/L dithiotritol, and 0.5% phenylmethyl sulfonyl fluoride, protease inhibitor cocktail (Hoffmann-La Roche) and phosphatase inhibitor tablets (PhosSTOP; Roche Diagnostics) and clarification by centrifugation at 15,000 rpm for 20 min at 4 °C. Protein samples containing comparable amounts of proteins, estimated by a modified Bradford assay (Bio-Rad), were resolved by sodium dodecyl sulfate-polyacrylamide gel electrophoresis (SDS-PAGE) gels and electrotransferred onto 0.2 µm nitrocellulose membranes (Trans-Blot Turbo; BioRad). After blocking membranes for 90 min at room temperature, they were incubated overnight at 4°C with primary antibodies, and then with a secondary antibody for 1 h at room temperature. Horseradish peroxidase-linked anti-rabbit (BioRad) and anti-mouse (BioRad) antibodies were used as secondary antibodies. Proteins were detected with Clarity Western ECL Substrate using the ChemiDoc (BioRad).  Images were analysed using BioRad software Image Lab 3.0.1.
